# The rationale for including immune checkpoint inhibition into multimodal primary treatment concepts of head and neck cancer

**DOI:** 10.1186/s41199-016-0009-6

**Published:** 2016-08-10

**Authors:** Ingeborg Tinhofer, Volker Budach, Korinna Jöhrens, Ulrich Keilholz

**Affiliations:** 1 Department of Radiooncology and Radiotherapy, Translational Radiation Oncology Research Laboratory, Charite University Hospital Berlin, Charitéplatz 1, 10117 Berlin, Germany; 2 2German Cancer Research Center (DKFZ), Heidelberg, and German Cancer Consortium (DKTK) partner site Berlin, Berlin, Germany; 3 Institute of Pathology, Charite University Hospital, Berlin, Germany; 4 Comprehensive Cancer Center, Charité University Hospital, Berlin, Germany

**Keywords:** Radiotherapy, Immune modulation, Combination therapy, Checkpoint inhibitor, Adaptive immunity

## Abstract

**Background:**

Treatment of locally advanced squamous cell carcinomas of the head and neck (SCCHN) remains unsatisfactory. Although the addition of concurrent radiochemotherapy (RCT) or the combination of radiotherapy with blockade of the epidermal growth factor receptor (EGFR) have improved outcomes over radiotherapy alone, further optimization is urgently needed. The introduction of immune checkpoint inhibitors is currently revolutionizing cancer treatment. Clinical evidence has recently been provided in melanoma that immune checkpoint blockade may cooperate with radiation. Therefore, we searched in the literature for the evidence of combining immune checkpoint inhibitors with radiotherapy in primary treatment of SCCHN.

**Discussion:**

A substantial amount of previous studies has dissected the molecular mechanisms of immune evasion in SCCHN. The biological effects of radio- and chemotherapy in tumor cells and the immune cell microenvironment were characterized in detail, revealing significant interference of both types of treatment with anti-tumor immunity. This extensive review of the literature revealed considerable amount of evidence that addition of immune checkpoint inhibitors might boost the immunomodulatory potential of radiotherapy and RCT regimens in SCCHN.

**Summary:**

Promising activity of immune checkpoint inhibitors has already been reported for metastatic/recurrent SCCHN. Given the immunogenic effect of radiotherapy and its enhancement by chemotherapy, combination of radiotherapy or RCT with this new type of immunotherapy might represent a valuable option for improvement of curative treatment modalities in SCCHN.

## Background

### Medical need for improvement of definitive treatment in locally advanced SCCHN

Patients with SCCHN completing radiotherapy-based treatment remain at considerable risk for local relapse within the radiation field, regional recurrence in the neck and hematogenous spread of tumor cells with the potential to form distant metastases. Primary radiochemotherapy (RCT) applied concurrently with cisplatin still cures less than 40 % of patients [1], and in case of disease recurrence after RCT, the 2-years survival rate is below 20 %. Furthermore, the addition of chemotherapy to radiation improves locoregional control at the cost of severe acute and late morbidity [2] but does not reduce the risk of distant metastases [1, 3].

During the last decade, there has been increasing interest in combining RCT with molecularly targeted agents. Most targeted approaches for radiosensitization tested so far have been directed against molecular pathways within cancer cells in order to increase the magnitude of DNA damage or to inhibit cellular mechanisms which interfere with tumor cell DNA repair, thereby increasing the efficacy of RCT. Based on the overexpression of EGFR in the majority of SCCHN cases and its causative role in radioresistance [4, 5] the EGFR signaling pathway was established as the first molecular target for radiosensitization in SCCHN [6, 7]. Consequently, the combination of cetuximab, a blocking antibody to EGFR with radiotherapy was shown to significantly improve outcome of locally advanced SCCHN when compared to radiotherapy alone [8]. However, despite improvement of locoregional control over radiotherapy alone, the cumulative rate of distant metastasis at 1 or 2 years remained unchanged by this combination [8]. Disappointingly, the RTOG study 0522 evaluating further treatment intensification by combining cetuximab with concurrent RCT failed to meet its endpoints to improve progression-free and overall survival [9]. Further trials which evaluated the combination of RCT with drugs directed against EGFR family members, a broader spectrum of receptor tyrosine kinases or the mTOR signaling pathway have not yet been completed or were stopped early due to the lack of significant activity (Table 1) which underlines the urgent need for novel concepts in this treatment setting. In view of the recent promising results of immune checkpoint inhibitors in the treatment of metastatic/recurrent SCCHN, combination of radiotherapy or RCT with this new type of immunotherapy might represent a valuable option. The aim of this review is to collect evidence from the literature which supports the notion that immune checkpoint blockade may cooperate with radiation in SCCHN.

**Table 1:** Clinical trials evaluating the combination of platinum-based RCT with targeted drugs in locally advanced SCCHN

Pathway/Target	Drug	Clinical trial	Results/Status
Tumor-specific targets (terminated trials)			
EGFR	Cetuximab	NCT00265941 (definitive, phase III, RTOG0522)	Negative
	Panitumumab	NCT00547157 (definitive, phase II, CONCERT-1)	Negative
	Cetuximab	NCT00791141 (adjuvant, phase II, ACCRA-HN)	Not yet reported
	Erlotinib	NCT00410826 (definitive, phase II)	Failed to significantly increase CRR or PFS
RTK (VEGFR2, EGFR, MET)	Vandetanib	NCT00720083 (adjuvant, phase II, RTOG0619)	Terminated early after 34 pts, no analysis
mTOR	Everolimus	NCT00858663 (definitive, phase I)	Terminated early, only assessment of outcome at 6 months - no responses seen
Tumor-specific targets (ongoing trials)			
DNA repair			
PARP	Olaparib	NCT02308072, (phase I, ORCA-2)	Recruiting
Cell cycle			
WEE-1	AZD1775	NCT02585973 (phase Ib)	Not yet recruiting
CHK-1	LY2606368	NCT02555644 (phase I)	Not yet recruiting
EGFR family			
EGFR/Her2	Lapatinib	NCT01711658 (phase II, TRYHARD)	Recruiting
AKT/PI3K			
PI3K alpha	BYL719	NCT02537223 (phase I)	Recruiting
Phospho-AKT	Nelfinavir	NCT02207439 (phase II)	Recruiting
Environmental targets (ongoing trials)			
Hypoxia	Nimorazole	NCT01880359 (phase III)	Recruiting
Immune checkpoints (ongoing trials)			
PD-1	Pembrolizumab	NCT02586207, (definitive RCT, phase I)	Recruiting
		NCT02641093 (adjuvant RT or RCT, phase II)	Recruiting
		NCT02296684 (adjuvant RCT, phase II)	Recruiting
CTLA-4	Ipilimumab	NCT01935921^a^ (definitive, phase I)	Recruiting
		NCT01860430^a^ (definitive, phase Ib)	Recruiting

^a^Ipilimumab combined with cetuximab-based bioradiation, not with platinum-based RCT

## Basic components of host immunity to cancer

In principle, the defense by the immune system against pathogenic microbes and toxins from the environment is divided into two general types of processes: the innate immunity and the adaptive immunity. Innate immunity recognizes and fights microbial invaders at the site of infection. In contrast, adaptive immunity is serving to eliminate host cells infected with viruses by recognizing peptides from intracellular viral proteins loaded onto major histocompatibility complex (MHC) molecules and displayed on the host cell surface. The adaptive immune system is also able to recognize mutated proteins in tumor cells via the same mechanism.

**Fig. 1:**
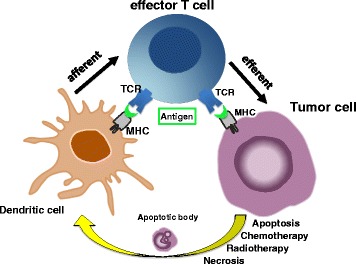
The afferent and efferent arms of adaptive tumor immunity. Tumor cells undergo apoptosis or necrosis, either spontaneously or after radio- or chemotherapy. Apoptotic bodies from tumor cells can be processed by dendritic cells. The antigen repertoire of dying cells is subsequently presented by dendritic cells via MHC molecules to T cells (the afferent arm of adaptive immune activation). T cells recognizing peptides by their T cell receptor (TCR) are activated and acquire cytolytic effector functions

There are multiple mechanisms by which a tumor cell harboring immunogenic mutations can elicit adaptive immune responses, as schematically summarized in Fig. 1. Tumor cells may spontaneously undergo apoptosis or necrosis, or may be driven to do so by radiotherapy and chemotherapy. The resulting apoptotic bodies can be processed by dendritic cells (DCs). The protein repertoire of dying cells is subsequently presented on the surface to T cells (the afferent arm of adaptive immune activation). T cells recognizing peptides derived from ‘foreign’ mutated proteins are activated by their and, after clonal expansion, these T cells search throughout the body for tumor cells displaying exactly this mutation on their surface. The cells which are recognized as carrying this mutation are killed through the lytic machinery of T cells (the execution of the efferent arm of adaptive immunity). However, in a patient with a growing tumor, this system has obviously failed as a consequence of one or many mechanisms which tumor cells have adopted to escape immune destruction.

### Immune evasion in SCCHN: hideout, defense, camouflage and balanced immune destruction

**Fig. 2:**
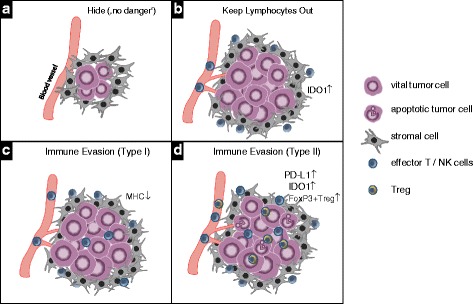
Mechanisms of immune evasion by tumors. **a** In the early phase of tumor development tumors remain undetected by the immune system because of the lack of danger signals such as significant levels of apoptotic or necrotic cells or pro-inflammatory cytokines. **b** By secretion of soluble factors such as indoleamine 2,3-dioxygenase (IDO) by tumor cells the infiltration of lymphocytes is inhibited. **c** If moderate immune cell infiltration eventually occurs tumor cells downregulate the expression of components of the antigen presentation machinery including MHC class I and II which results in their impaired recognition by antigen-specific T cells. **d** In tumors with a larger extent of immune cell infiltration, tumor cell destruction by cytotoxic T cells is inhibited by high expression of immunosuppressive mechanisms such as IDO, PD-L1 and FoxP3+ Treg

**Fig. 3:**
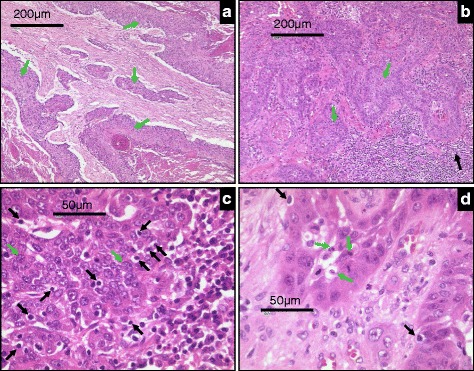
Representative histological images of SCCHN tumor sections displaying different levels of immune evasion. **a** Tumor areas (*green arrows*) show the absence of any lymphocyte filtration at the rim or within the tumor cell nests. **b** Lymphocyte infiltrates are seen at the tumor border (*black arrows*) but are absent within the tumor nests (*green arrows*). **c** Despite a high extent of lymphocyte infiltration no signs of tumor cell lysis or apoptosis are visible. **d** Tumor areas with infiltrating lymphocytes are composed of vital and apoptotic tumor cells (*black arrows*), indicative of a balance between immune destruction and evasion

There are several ways for SCCHN to evade recognition by the adaptive immune system, as schematically illustrated in Fig. 2. Early tumors may grow in a hideout, because they display neither a significant level of apoptosis or necrosis nor inflammatory signals and thus do not elicit any danger signal in the tissue (Fig. 2a). Although danger signals might subsequently be produced during the progression of the disease, the lymphocytic infiltrate may be confined to the rim of the tumor tissue with no infiltration into the tumor itself (Fig. 2b). Secretion of indoleamine 2, 3-dioxygenase (IDO) is among the major defense mechanisms used by tumors to prevent lymphocytic infiltration [10]. Tumors frequently also counterbalance infiltration by lymphocytes by down-regulation of their MHC molecules, thereby avoiding the presentation of peptides from intracellular proteins to T cells which results in an effective camouflage (Fig. 2c). As schematically depicted in Fig. 2d, in tumors with extensive inflammatory cell infiltration a delicate balance between immune destruction and immune evasion may exist which is based on immunosuppressive mechanisms including high expression of IDO and PD-L1 as well as recruitment of FoxP3+ regulatory T cells (Treg) [11]. Representative histological images from SCCHN tumor sections exemplifying the above-mentioned types of immune evasion are presented in Fig. 3.

A number of both genetic and environmental mechanisms allow such immune escape and have been described in SCCHN (for a recent detailed review see [12]), including selection of poorly antigenic cancer cell subsets, disturbances in MHC class I- and class II-mediated antigen presentation [13–15], expression and secretion of immunosuppressive cytokines [16], expression of the pro-apoptotic Fas ligand to induce activation-induced cell death in T cells [17], and recruitment of immunosuppressive immune cell subsets into the tumor [18]. More recently, evidence is increasing that expression of immune checkpoint components that may limit T cell responses also occurs frequently in these tumors [18–20].

## Immunomodulatory effects of ionizing radiation

Ionizing radiation has been used for more than a century to treat cancer [21], on the basis that rapidly proliferating cancer cells are more sensitive to DNA damage induced by radiation than normal tissue. Radiotherapy has traditionally been viewed as immunosuppressive due to the inherent sensitivity of lymphocytes to radiation-induced damage but it became evident that radiotherapy can also enhance tumor-specific immune responses. Strong support of an active role of the immune system for the success of radiotherapy came from studies in which the extent of tumor control by radiotherapy was compared in immunocompetent and -deficient xenograft models. Studies in the model of melanoma revealed that the ablative effects of radiotherapy were strongly dependent on radiation-induced cytokine responses [22] and cytotoxic CD8+ T cells [23]. In a preclinical model of SCCHN, pretreatment of tumor cell lines with chemotherapeutic drugs and radiation significantly increased the extent of their cytolysis by antigen-specific CD8+ T cells [24]. All these observations suggest that not only genetic and phenotypic traits of tumor cells but also immunity of the host are implicated into the clinical success of radiotherapy [22, 23, 25–27].

Mechanistically, radiotherapy has been shown to augment the afferent as well as efferent arms of cancer immunity. The induction of a specific T cell response to tumor cells (afferent immunity) has been observed in multiple studies. Almost 20 years ago molecular pathways were first described that were activated by treatment-induced cell stress (in particular after treatment with anthracyclins and ionizing radiation) and which induced a modality of cell death that was highly efficient in eliciting immune responses [28]. The immunogenic effects of radiation (reviewed in [29]) are thought to result from ‘autovaccination’ by antigens released from dying tumor cells. Translocation of a protein called calreticulin which is normally residing in the endoplasmatic reticulum to the cell surface promotes the uptake of dying cancer cells by DCs and the release of antigens that can be efficiently presented [30]. Release of ATP, heat shock proteins and high-mobility group box 1 (HMGB1) by dying cancer cells help in recruiting and activating DCs through toll-like receptor signaling pathways [31]. By integration of these danger signals DCs undergo an important maturation process. They upregulate the expression of co-stimulatory proteins and pro-inflammatory cytokines, and acquire the ability of cross-presenting antigens to cytotoxic CD8+ T cells by which they initiate adaptive immunity [32].

Radiotherapy can also influence the efficacy of tumor cell destruction (efferent immunity) within the radiation field by altering tumor cell characteristics or the tissue microenvironment. Tumor cells in which the damage from radiation has not been sufficient to induce cell death show increased expression of MHC class-I antigen-presenting molecules [33] and adhesion molecules [34], stabilizing the binding of T cells to tumor cells and alleviating TCR activation. As a result, tumor cells that survive radiation may be eliminated through CD8+ T cell-mediated lysis [33].

It has been known for some time that irradiated tissues often show very strong changes in the local cytokine milieu. As a result of their action, a cascade of pro-inflammatory processes are triggered. Secretion of interferon-γ enhances expression of MHC class-I by cancer cells, sustaining and extending the initial effects of radiation to allow efficient recognition and killing by T cells [26, 34]. In addition, immune cell trafficking is enhanced through induction of chemokines, such as CXCL16 that attract effector T cells to the irradiated tumor site [29].

Immune effects within the radiation field, however, are not sufficient for cure, as effective treatment of a high-risk primary cancer has to secure not only local but also systemic control of the disease. In principle, the nature of the adaptive immune system should be mechanistically well suited for systemic tumor surveillance. There is emerging evidence that radiotherapy can also be associated with immune destruction of distant metastases, pointing towards efferent immunity outside of the radiotherapy field. This phenomenon termed abscopal anti-tumor effect was already described in 1953 [35]. Clinical reports of an abscopal effect after radiotherapy are few, but cover several different tumor types, including melanoma and a variety of carcinomas [36]. Growth suppression of distant non-irradiated tumors by a combination therapy of DC infusion and radiotherapy was also reported in a murine model of squamous cell carcinoma in the head and neck, indicating that indeed systemic antitumor activity can be induced by approaches which augment the immune-activating effects of radiotherapy.

The knowledge gained from mechanistic studies on the immunomodulatory effects of radiation mentioned above has changed the way the response to radiotherapy with/without chemotherapy in patients with cancer is now interpreted, by acknowledging the essential role of the host immune system for the success of radiotherapy. Importantly, these indirect effects of radiation – within and outside the field of treatment – also suggest new treatment possibilities, including combinations with established or novel forms of immunotherapy.

## Immune checkpoint blockade as novel immunotherapeutic strategy in cancer

**Fig. 4:**
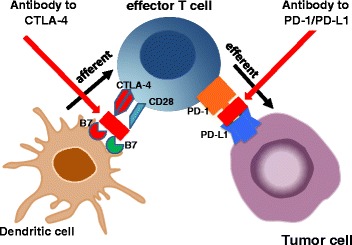
Immune checkpoints as modulators of the afferent and efferent arm of adaptive immunity. Cytotoxic T-lymphocyte protein 4 (CTLA-4) is an inhibitory receptor acting as a major negative regulator of T cell responses. As part of the afferent immune response CTLA-4 upregulation on antigen-activated T cells dampens the magnitude of T cell activation. At the efferent side, programmed death receptor 1 (PD-1) which is expressed on activated T cells blocks their effector functions upon binding to the ligands PD-L1 or PD-L2 on target cells. Tumor cells frequently use the expression of PD-L1 and PD-L2 to escape immune destruction

The introduction of immune checkpoint inhibitors is currently revolutionizing treatment of metastatic cancers. Previously, cancer immunology had concentrated either on afferent immune stimulation, i.e. induction of T cell immunity, most frequently by vaccination, or on stimulation of efferent T cell activity, e.g. by interleukin-2 treatment. An important limitation of these approaches was the tight regulation of the immune system by mechanisms termed immune checkpoints which are physiologically crucial to prevent autoimmune diseases (Fig. 4). At the afferent side of immunity the molecule cytotoxic T-lymphocyte protein 4 (CTLA-4) is expressed on antigen-activated T cells to dampen the magnitude of T cell activation. At the efferent side, the expression of the cell surface receptor PD-1 (programmed cell death protein 1) on activated T cells block their effector function, if bound to the ligand PD-L1 or PD-L2 on the target cell. Tumor cells frequently use the expression of PD-L1 and PD-L2 to escape immune destruction. Blocking antibodies directed to the immuno-regulatory proteins CTLA-4, PD-1 and PD-L1 have been shown to release these immune checkpoints in different ways. Antibodies to CTLA-4 (namely ipilimumab and tremelimumab) allow induction of autoimmunity, including immunity to cancer. However, there is a tight window of opportunity, as autoimmune phenomena can be quite serious after application of these agents [37]. Antibodies to PD-1 or PD-L1 do not promote induction of *de-novo* immunity but release the effector phase of immunity (Fig. 4), hereby allowing the execution of tumor cell destruction by T cells. Thus, the presence of tumor-specific T cells is required for efficacy of agents interfering with the PD-1/PD-L1 interaction.

The application of immune checkpoint inhibitors has recently been evaluated in a number of clinical trials and demonstrated remarkable activity in a broad spectrum of cancer types. Ipilimumab, nivolumab and pembrolizumab (the latter two agents both anti-PD-1 antibodies) were the first three immune checkpoint inhibitors which received FDA approval for the treatment of metastatic melanoma. A three-arm phase III trial in melanoma [38] answered the fundamental question in cancer immunology as to whether the *de-novo* induction of T cell responses by ipilimumab or the augmentation of a pre-existing T cell response by nivolumab may be more efficacious. Response rates and progression-free survival clearly favored nivolumab over ipilimumab, with the combination of both even more effective but at the cost of considerable immune-related toxicities [38].

There are at least eight anti-PD-1/PD-L1 antibodies currently in clinical development, covering phases I to III. In addition, the preclinical and early clinical development of inhibitors against other immune checkpoints, such as T cell immunoglobulin mucin receptor 3 (TIM3) and lymphocyte activation gene 3 protein (LAG3), and against co-stimulatory molecules, such as OX40 and CD137, are underway. Final results from several successful phase III trials with ipilimumab, nivolumab and pembrolizumab improving overall survival of metastatic cancer have been reported in melanoma and lung cancer, and it can be expected from the data available for a broad range of other histologies that this novel class of agents will be firmly established in modern treatment of many cancers.

In recurrent/metastatic SCCHN, several PD-1/PD-L1 blocking agents are currently being investigated, with most mature information on nivolumab and pembrolizumab. The phase 1b multicohort trial Keynote-012 tested the efficacy of the anti-PD-1 antibody pembrolizumab for treatment of PD-L1+ in recurrent/metastatic SCCHN [39]. A best overall response rate of 18 % was reported, with no obvious difference being observed between HPV+ (25 %) and HPV- tumors (14 %). Duration of responses was approximately 12 months [39]. Comparable results (overall response rate: 18 %; HPV+, 22 %; HPV-, 16 %) were reported for the Keynote-055 study in patients with R/M SCCHN resistant to platinum and cetuximab have been included [40]. Moreover, the randomized global phase III trial Checkmate-141, evaluating the efficacy and safety of nivolumab versus investigator’s choice in patients with R/M SCCHN demonstrated an increase in 1-year overall survival (OS) rate from 16 to 36 % by nivolumab [41, 42]. Again, a survival benefit was observed in the HPV+ and HPV- subgroup [41, 42].

Early evidence of clinical activity in SCCHN were also reported from multi-arm expansion studies of anti-PD-L1 antibodies (atezolizumab, MPDL3280A [43]; durvalumab, MEDI4736 [44]). Based on these promising data, several further randomized phase III trials (NCT02358031, Keynote-048; NCT02252042, Keynote-040) have been initiated. In general, the successful clinical trials of PD-1 blocking agents are a proof of the existence of adaptive immunity towards SCCHN cells which can be very effective in a proportion of patients when unleashed by blockade of the PD-1/PD-L1 interaction.

### Interference of immune checkpoints with resistance to RCT

Deregulated expression of immune checkpoint proteins has already been linked to poor efficacy of RCT in several tumor models. High expression of PD-L1 in tumor cells and stromal lymphocytes accompanied by low CD8+ T cell infiltration has recently been identified as a poor prognostic biomarker in patients with stage III non-small cell lung cancer (NSCLC) receiving cisplatin-based RCT [45]. In addition, tumor control by neoadjuvant or concurrent RCT was observed to be inefficient in patients with esophageal squamous cell carcinomas displaying elevated immunostaining for PD-L1 in neoplastic and adjacent non-malignant esophageal epithelium [46]. Preclinical studies in a variety of syngeneic mouse models of cancer [47] have also demonstrated that expression of PD-L1 can be induced by radiation itself and that such upregulation impairs both local tumor control and protection against tumor re-challenge [47]. It is therefore not surprising that blocking antibodies directed to these immune checkpoints were able to significantly enhance the immunogenic effects of radiotherapy [27, 48, 49].

In locally advanced SCCHN the magnitude of immune suppression could also be linked to the efficacy of RCT, however, a direct role of immune checkpoint proteins remains to be established. Low numbers of tumor-infiltrating cytotoxic CD8+ T cells before treatment were significantly correlated with poor outcome after RCT in several studies [50–53] but the role of CTLA-4 or PD-1/PD-L1 as negative regulators of CD8+ T cell activation has not been addressed. By inducing a change in the composition and functions of the immune cell compartment RCT was shown to relieve the extent of immune suppression: while the numbers of CD8+ and granzyme B+ cytotoxic cells only slightly decreased after RCT, a more pronounced decrease of FoxP3+ Treg was observed, resulting in an 2- to 3-fold increase in the cytotoxic T cell/FoxP3+ Treg ratio [50]. These data strongly support the idea that application of immune checkpoint inhibitors together with radio(chemo)therapy could also lead to a significant improvement of local as well as distant tumor control in SCCHN. Consequently, the first phase I/II trials evaluating the combination of pembrolizumab with standard definitive or adjuvant RCT as well as ipilimumab with cetuximab-based bioradiation (Table 2) have already been started, and several further trials with other inhibitors of PD-1 or PD-L1 are in preparation.

**Table 2:** Current clinical trials (at clinicaltrials.gov) evaluating the combination of RT with immune checkpoint inhibitors

Clinical setting	Clinical trial	Drug	Combination
Resectable locally advanced SCCHN	NCT02641093 phase II	Pembrolizumab	Adjuvant RT/RCT
	NCT02296684 phase II	Pembrolizumab	Adjuvant RT/RCT
Locally advanced SCCHN	NCT01935921 phase I	Ipilimumab	Definitive RT + cetuximab
Intermediate/High risk locally advanced SCCHN	NCT01860430 phase Ib	Ipilimumab	Definitive RT + cetuximab
Locally advanced SCCHN	NCT02586207 phase I	Pembrolizumab	Definitive RT + CDDP
locally advanced laryngeal carcinoma	NCT02759575 phase I/II	Pembrolizumab	Definitive RT + CDDP
Intermediate/High risk locally advanced SCCHN	NCT02764593 phase I	Nivolumab	Definitive RT, RT+ CDDP, RT + cetuximab
	Phase III	Nivolumab	Definitive RT + CDDP
locoregional inoperable recurrence or second primary SCCHN	NCT02289209 phase II	Pembrolizumab	Reirradiation
Advanced metastatic disease (multicohort)	NCT02303990 phase I	Pembrolizumab	RT
Brain metastasis (multicohort)	NCT02669914 phase II	Durvalumab	Stereotactic radiosurgery

*RT* radiotherapy, *CDDP* cisplatin

### Potential biomarkers for patient selection for immune checkpoint blockade

Precise biomarkers to identify patients who benefit from immune checkpoint inhibition alone or in combination with RCT still have to be established. Current data in multiple cancers reveal that verification of PD-L1 overexpression by immunohistochemistry is associated with improved clinical outcome of anti-PD-1 therapy. However, the presence of robust responses in some patients with low or undetectable expression of PD-L1 complicates the issue of PD-L1 as an exclusive predictive biomarker [54]. In the Keynote-012 trial of SCCHN, an elevated expression of PD-L1 and the presence of an interferon-γ expression signature were associated with improved progression-free survival [39, 55]. The same signature had previously been established as predictive signature for outcome after pembrolizumab in metastatic melanoma [56] and its predictive value was also demonstrated in advanced gastric cancer [57]. In addition, patients with large immune cell infiltration in tumor tissue and high mutational load were more likely to benefit from immune checkpoint blockade in bladder [58] and colorectal cancer [59]. Taken together, patients with PD-L1+ tumors displaying immune-related gene expression signatures, including genes regulating T cell functions, the antigen presentation machinery and IFN-γ signaling, are most likely to benefit from immune checkpoint inhibition.

### Rational development of combination regimens

While there is much excitement around the phenomenon of a radiation-induced anticancer immune response and combining radio(chemo)therapy with immunotherapy, numerous questions remain to be addressed in clinical trials. A major challenge is to identify not only the optimal immune checkpoint inhibitor as partner for a given radiotherapy schedule but also the best chronological sequence for their combined application. Preclinical evidence can serve as guidance in treatment schedule and clinical trial development. As outlined above, danger signals induced by radiation lead to the recruitment of immune cells into the tumor. However, cells of the immune system are also vulnerable to radiation, as their exposure to ionizing radiation induces apoptosis in mature natural killer (NK) cells as well as T and B cells. Since radiotherapy is generally delivered in daily fractions, re-irradiation of the tumor might therefore damage infiltrating immune cells that display cytolytic activity themselves or might significantly reduce the capacity of DCs to activate effector T cells. In addition, DCs may find a hostile environment for T cell activation in draining lymph nodes, which represent their natural surrounding for interaction with T cells, as draining lymph nodes are systematically included into the radiation field in SCCHN. Conversely, however, the induction of immunogenic cell death (ICD) by each daily radiotherapy fraction might transiently generate a favorable milieu for immune activation within the tumor tissue, which may vanish again after termination of radiotherapy. In support of the latter, concurrent application of an anti-PD-L1 antibody together with fractionated radiotherapy significantly improved tumor control in a xenograft model [47]. In contrast, fractionated radiotherapy followed by delayed application of anti-PD-L1 was completely ineffective in enhancing the local efficacy of radiotherapy [47]. Certainly, more studies will be needed to address this important issue.

Besides the optimal time schedule also the optimal type and dosing of chemotherapy has to be established, if desired to be included into the treatment regimen. Significant differences in the ability of chemotherapeutic drugs to induce ICD have been reported previously [32]. Cisplatin which is an essential component of current state-of-the-art RCT regimens in SCCHN does not induce ICD [60] despite its presumed identical mechanism of action to that of oxaliplatin, a potent inducer of ICD. This has been attributed to the lack of calreticulin exposure after cisplatin treatment [60]. However, radiotherapy is a potent inducer of calreticulin exposure [30], and recent studies have shown that combining cisplatin with compounds that induce calreticulin exposure leads to full-scale ICD [60]. Thus, potentiation of ICD by cisplatin could still represent one of its major mechanism of action when cisplatin is administered concurrently with radiotherapy.

Taxanes including docetaxel and paclitaxel which are also common combination partners of radiotherapy in locally advanced SCCHN are known to modulate antitumor immune responses as well [61]. Similar to cisplatin, paclitaxel does not induce ICD. However, concurrent paclitaxel treatment was shown to significantly enhance radiation-induced ICD in breast cancer cell lines [62]. Similarly, docetaxel treatment itself did not induce ATP or HMGB1 secretion by tumor cells. However, calreticulin exposure of tumor cells after docetaxel treatment was observed which significantly enhanced tumor cell killing by antigen-specific CD8+ cytotoxic T cells [61].

Intriguingly, investigations on the immunogenic effects of chemotherapeutic drugs revealed also their direct interference with immune checkpoint expression. Treatment of DCs in vitro with platinum-based compounds strongly enhanced their potential to activate T cells which was caused by downregulation of PD-L2 in DCs [63]. This effect was mediated by inactivation of STAT6, the transcriptional regulator of PD-L2, and occurred also in tumor cells resulting in their enhanced recognition by T cells [63].

Overall, these preclinical observations provide a sound rationale for investigating immune checkpoint inhibitors with radiotherapy alone as well as in combination with standard cisplatin-based as well as taxane-based RCT. In addition, there is accumulating data that the efficacy of cetuximab-based regimens in treatment of recurrent/metastatic SCCHN is not only based on the inhibition of EGFR signaling pathways but also on the activation of Fcγ receptor-positive NK cells leading to DC maturation and activation of cytotoxic T cells [64]. A combination of immune checkpoint inhibitors with cetuximab-based bioradiation protocols might therefore also represent a very attractive chemotherapy-free concept for improvement of primary treatment of locally advanced SCCHN.

### Toxicity of combination regimens

The toxicity of radiotherapy is mostly occurring directly at the irradiation site. Mucositis, xerostomia and swallowing dysfunctions are common side effects in radiotherapy of head and neck cancers. Clearly, the extent of early and late toxicity is dependent on the radiation technique and the applied dose: Intensity-modulated radiotherapy, which conforms closely to the tumor volume, avoids or minimizes exposure to unaffected tissue and thereby significantly reduces local side effects of irradiation [65]. On the other hand, addition of concurrent chemotherapy to radiotherapy not only improved efficacy of the treatment in locally advanced SCCHN but also increased both the toxicity and the spectrum of adverse events as compared to radiotherapy alone [66]. The toxicity of immunotherapy is dependent on the administered agent and dosage. In previous clinical trials, immune checkpoint blockade immunotherapy presented acceptable toxicity. Even occasional severe toxicity was manageable through treatment interruption or involvement of immunosuppressive drugs. During ipilimumab treatment approximately 60 % patients showed immune-related adverse events, of them 10–15 % being grade 3–4 [67]. The blockade of PD-1/-L1 showed less severe ir-AEs in previous phase I studies [68]. Diarrhea and skin rash were the most common immune-related adverse events after ipilimumab, other adverse effects included enterocolitis, hypothyroidism, hypophysitis and neuropathies [68]. The most common adverse events reported for both nivolumab and pembrolizumab were mild fatigue, rash, pruritus and diarrhea, which could be usually managed without dose interruption or discontinuation [68]. First toxicity data from a phase I study of the combined application of ipilimumab with radiotherapy for treatment of metastatic melanoma (NCT01497808, [48]) argue against an exacerbating toxicity profile of the combined regimen. No dose-limiting toxicities, defined by the study protocol as any treatment-related grade ≥4 immune-related toxicity or grade ≥3 non-immune related toxicity experienced during study treatment or within 30 days after the last injection of ipilimumab were observed [48]. Considering the different kinds and acceptable adverse events, the combinatorial treatment of radiotherapy and immune checkpoint inhibitors seems feasible for SCCHN patients.

## Conclusions

The introduction of immune checkpoint inhibitors into cancer treatment has been celebrated as the breakthrough of the year 2013. Impressive activity was proven in metastatic melanoma and lung cancer, and promising results were presented for recurrent/metastatic SCCHN. Given the immunogenic effect of radiotherapy and its enhancement by chemotherapy or cetuximab, it remains to be determined whether immune checkpoint inhibitors could further increase the activation of adaptive immunity and ultimately improve overall current cure rates of locally advanced SCCHN.

## Abbreviations

CTLA-4, cytotoxic T-lymphocyte protein 4; DCs: dendritic cells; DNA, deoxyribonucleic acid; EGFR, epidermal growth factor receptor; HMGB1, high-mobility group box 1; ICD, immunogenic cell death; IDO, indoleamine 2:3-dioxygenase; LAG3, lymphocyte activation gene 3 protein; MHC, major histocompatibility complex; NK, natural killer; NSCLC, non-small cell lung cancer; PD-1, programmed cell death receptor 1; PD-L1, programmed cell death 1 ligand 1; RCT, radiochemotherapy; SCCHN, squamous cell carcinoma of the head and neck; TCR, T cell receptor; TIM3, T cell immunoglobulin mucin receptor 3; Treg, regulatory T cells
